# Advances of 3D microcrystals electron diffraction for transmembrane protein structure determination

**DOI:** 10.1016/j.bpj.2025.10.027

**Published:** 2025-10-23

**Authors:** Giorgia Fiorini, Gebhard F.X. Schertler, Valérie Panneels

**Affiliations:** 1 Division of Biology and Chemistry, Laboratory for Biomolecular Research, Paul Scherrer Institute, Villigen PSI, Switzerland

## Abstract

Electron diffraction of 3D microcrystals (3D ED/MicroED) is an emerging technique that uses electrons to determine proteins structure from their nano-sized 3D crystals, thus overcoming the limitations on crystal-size dimensions generally imposed by x-ray or neutron diffraction. The strong interactions of electrons with matter have the potential to reveal the location of light atoms (e.g., hydrogens), revealing interaction networks, and to provide information about the charge states of the atoms. Recent advances in MicroED sample preparation and data collection strategies have led to structural determination of several known and unknown protein structures, including challenging targets such as membrane proteins. In this review, we provide an overview of the recent advances of MicroED including sample preparation and data collection.

## Significance

Determining the 3D structures of proteins is essential for understanding how they function. However, proteins structural biology is challenging, and the currently available methods used for structural studies have many limitations. Recent advances in a relatively new method, microcrystal electron diffraction (MicroED), have the potential to broaden the range of accessible targets for structural study. MicroED not only makes previously inaccessible proteins more approachable, but it also offers unique advantages, such as detecting hydrogen atoms and atomic charge states. This review highlights some of the key recent advances in sample preparation and data collection that are driving the rapid progress of MicroED, making it an increasingly powerful tool for structural biology.

## Introduction

Microcrystal electron diffraction (MicroED) is gaining recognition as a method for studying protein structures, complementing established techniques such as x-ray diffraction and cryo-electron microscopy (cryo-EM) (1,2,3). MicroED uses an electron beam that is diffracted by samples in their crystalline (or powder) form (2). Like x-ray diffraction and cryo-EM, MicroED enables structural determination of isolated proteins. Notably, ongoing developments in sample preparation and image analysis have expanded the use of cryo-electron tomography (cryo-ET) for in situ structural studies (4).

Unlike x-rays, which interact primarily with the electron cloud of the atoms, electrons are scattered by both the electron cloud and nuclei of the atoms (1). Hence, the stronger interaction using electrons enables structural determination from relatively smaller crystals than those required for x-ray diffraction (1), and it has the potential to localize light atoms (e.g., hydrogens) and provide information about the atoms charge states (5,6,7). However, it has the inconvenience to statistically produce multiple scattering events, usually negligible in x-ray diffraction (8), that affect electron diffraction data. Statistical corrections and data collection strategies can help reduce the impact of multiple scattering (7,8,9).

Differently from MicroED and x-ray diffraction, cryo-EM exploits high-magnification imaging to capture 2D projections of randomly oriented particles in their near-native state (2). Cryo-EM has been particularly advantageous for investigating large protein complexes whose structures were still elusive (10). Currently, one of the main limitations of cryo-EM remains the difficulty of achieving atomic-level resolution for relatively small molecular complexes (<40 kDa) (2,11).

The first biological structures, solved using a combination of electron diffraction and imaging, were the transmembrane alpha helices in the purple membrane (12) and the light-harvesting chlorophyll a/b-protein complex (LHC-II) (13,14), showcasing the early application of electron diffraction to 2D crystals (12,14). Advances in the field have since enabled the transition to electron diffraction from 2D to 3D crystals, broadening the range of targets accessible to structural study. MicroED offers a complementary method for structural studies and a valuable alternative to x-ray diffraction and cryo-EM studies, when crystallization produces small crystals that are hard to optimize further, and when cryo-EM is not a possibility (e.g., low-resolution images, low molecular weight of the target).

Recent advances in x-ray diffraction have also enabled to decrease the minimum sample dimensions for data collection (15,16,17). The recent development of micro/nano-focus macromolecular crystallography beamlines (e.g., the VMXm (16) at Diamond Light Source) provides the opportunity to perform structural studies on submicron-sized crystals deposited on EM grids. Other micro- and nano-focused beamlines for diffraction experiments are also available at the European Synchrotron (ESRF, ID13) (18), Swiss Light Source (microXAS) (19), or at the MAX IV Synchrotron (NanoMAX) (20). X-ray free electron lasers (XFELs), which use bright micro-focused x-ray pulses (21,22), also enable data collection from nano-/micro-sized crystals. Importantly, due to the high intensity of the pulse used at XFELs, crystals are destroyed upon exposure to the beam (22,23). As a result, images collected from thousands of individual crystals have to be merged to obtain a complete data set (22), leading to high sample consumption. Unlike x-rays, electrons are less damaging to the sample (24), thus enabling multiple exposures of a single nanocrystal (17).

Another advantage is that MicroED is performed on transmission electron microscopes (TEMs) or purpose-built electron diffractometers (Eldico Scientific or Rigaku) (25,26,27) that are generally less expensive to maintain than XFELs (23). By contrast, the use of nano-focused beams requires access to specialized synchrotrons or XFELs, which may be limiting.

**Figure 1:**
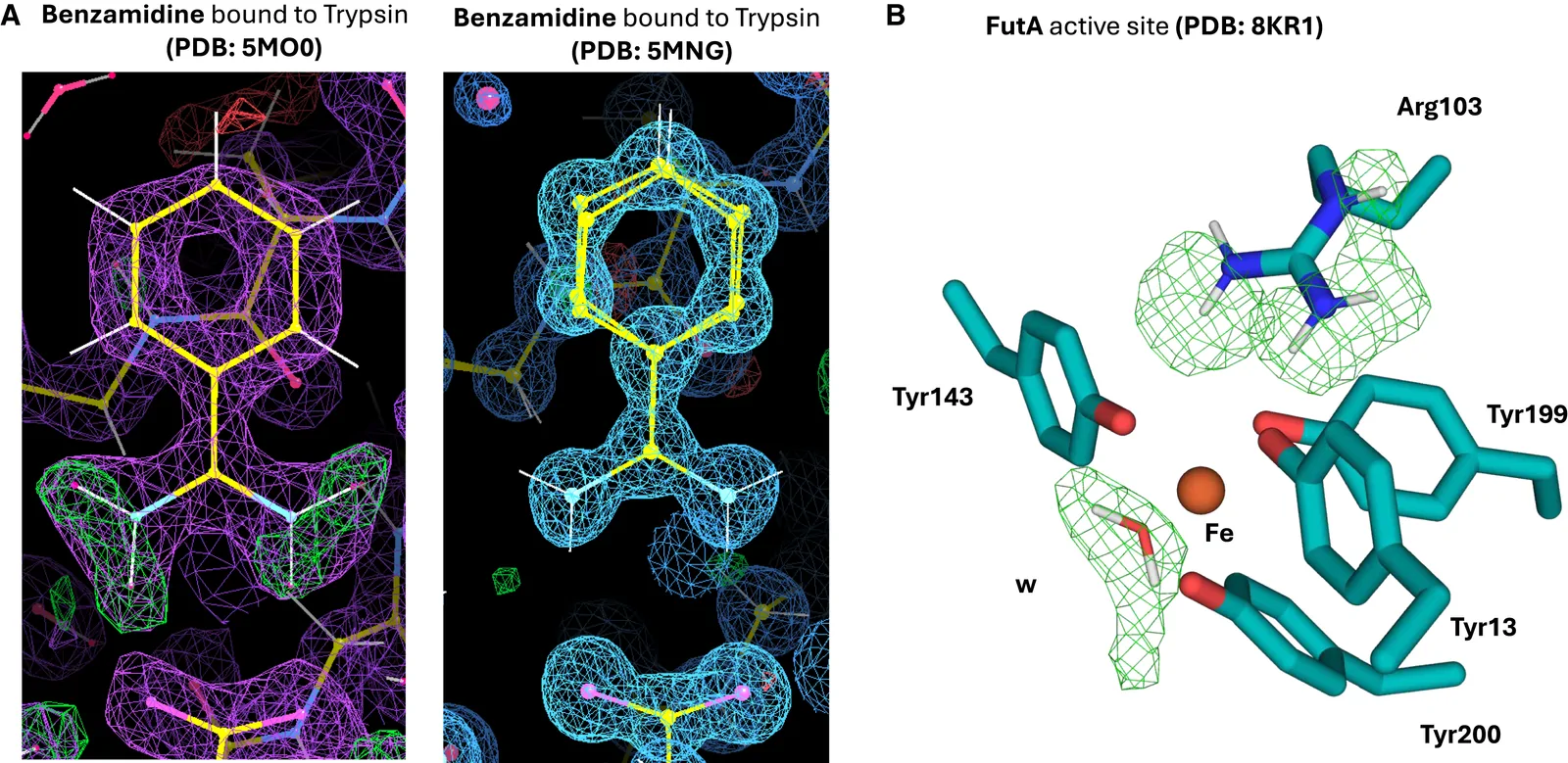
Structural insight from neutron diffraction. (*A*) Close-up view of benzamidine in complex with trypsin from structures solved by neutron diffraction (PDB: 5MO0) and x-ray diffraction at 100 K (PDB: 5MNG) (30). (*B*) View of the active site residues of the iron-binding protein FutA and neutron mFo-DFc map showing hydrogen locations on a water molecule and Arg103 residue (PDB: 8KR1) enabling determination of the Fe oxidation state (29). (*A*) Neutron and x-ray 2mFo-DFc maps are contoured to 1.8σ as purple and light blue meshes, respectively. Neutron mFo-DFc omit maps are shown as green mesh contoured to 3σ. The figure was made in Coot (31). (*B*) The neutron mFo-DFc map is contoured to 2.5σ as green mesh. The figure was made in PyMOL. Fe, orange sphere; active site residues, dark cyan sticks; water, red and white sticks; hydrogen, white sticks.

In an era in which protein structure prediction can be easily performed in silico, the value of structural determination, through experimental methods, lies in the possibility to uncover details of protein structures and mechanisms that would otherwise remain inaccessible. Such details include insights on protonation states and hydrogen bonds in proteins, ligand recognition (28,29), or catalytic intermediates forming during enzymatic reactions. Interesting examples, coming from neutron diffraction studies, show the importance of revealing hydrogen location to understand the exact geometry of hydrogen bonds between proteins and their inhibitors (30) (Fig. 1 *A*) or to reveal the oxidation states of metal centers in proteins (29) (Fig. 1 *B*).

Similarly, MicroED holds great promise as it overcomes key limitations of existing experimental techniques, and it has been shown to be able to successfully resolve atomistic details, including hydrogen bonding and atoms charge states in proteins (7,32). Beyond proteins, MicroED finds important applications in small organic molecules, pharmaceutical science, and materials science (9,33,34,35,36), and it has recently been implemented in natural products discovery workflows (37). Currently, data coming from small molecules outnumber protein structures in the Protein Data Bank (PDB) and in the Cambridge Structural Database (25). Despite its advantages (Table 1), MicroED is in its early development for protein studies, and further work is needed to improve its accessibility. In this review, we will summarize the recent advances in protein MicroED data collection and sample preparation that have enabled structural studies of numerous proteins and, among them, membrane proteins.

**Table 1:** Summary of some key characteristics of X-ray crystallography, MicroED, Cryo-EM, Neutron diffraction, and Cryo-ET

Technique	X-ray diffraction	MicroED	Cryo-EM	Neutron diffraction	Cryo-ET
Source	X-ray	Electrons	Electrons	Neutrons	Electrons
Data collection mode	Diffraction	Diffraction	Imaging	Diffraction	Imaging
Device	Synchrotrons/XFELs	TEM	TEM	Neutron source	TEM
Achievable resolution	Atomic level	Atomic level Can reveal light atoms locations and charge states	Near-atomic level for protein complexes >40 kDa	Atomic level Can reveal light atoms locations and charge states	Subnanometer resolution (when averaging is applied)
Limitations	High sample concentrations Large complexes can be hard to crystallize Expensive facilities	Multiple scattering events Thin sample required Still not fully automated	Hard to image protein complexes <40 kDa	High sample concentrations/large crystals Large complexes can be hard to crystallize Expensive facilities	Hard to image protein complexes <500 kDa Low throughput Challenging sample preparation

Note that this summary is not extensive.

The information reported in this table is discussed in the following references: X-ray crystallography (15,22), MicroED (38), Cryo-EM (11), Neutron diffraction (39), and Cryo-ET (4,40).

## Advances in preparation of biological samples for MicroED

MicroED requires submicrometer-thick crystals to be vitrified on EM grids (41). The vitrification step is generally preceded by application of the crystal slurry on the grid, followed by blotting of the excess liquid and plunge-freezing into liquid ethane. The ice surrounding the sample needs to be thin enough to optimize signal/noise ratio while still maintaining the hydrated state of the proteins (41). Notably, recent studies showed that it is possible to obtain and index MicroED diffraction data from non-vitrified lysozyme nanocrystals (42). To maintain the hydrated state of the crystals under the TEM vacuum, graphene liquid cells were used to trap the nanocrystals in their mother liquor (42).

To date, specimen preparation is still one of the major bottlenecks of MicroED (41), and several aspects require optimization: crystal size, EM grid type, transfer of the crystals onto the EM grid, blotting time, force, humidity, and temperature. Although the optimal blotting/vitrification conditions are sample specific, in their recent review, Gonen et al. (43) provide an extensive explanation on how to perform and optimize sample preparation.

**Figure 2:**
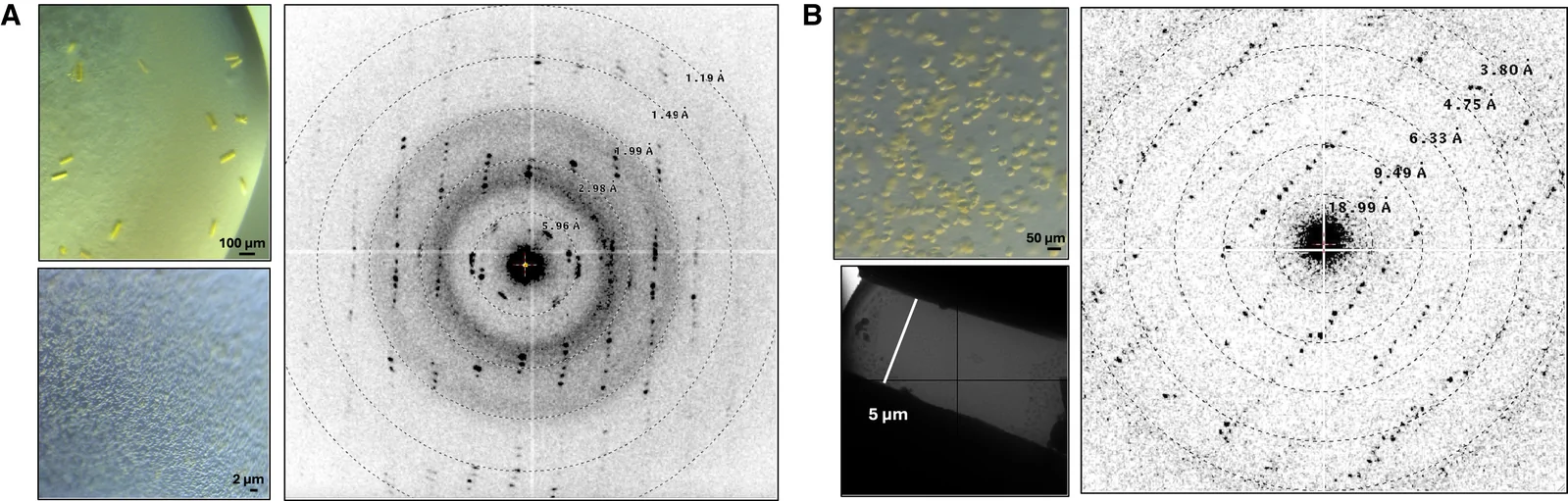
MicroED images of Cr-PhotLOV1. (*A*) Image of crystals of the light-oxygen voltage (LOV) domain of phototropin Phot-1 from *C. reinhardtii* (Cr-PhotLOV1) obtained with no seeding (*top left*) (48). Scale bar: 100 μm. Image of crystals of Cr-PhotLOV1 after seeding (*bottom left*), scale bar: 2 μm ,and electron diffraction image obtained from bottom left crystals on a JEM F200 at 200 kV equipped with energy filter (1.4-μm beam size, 700-mm detector distance). (*B*) Image of crystals of Cr-PhotLOV1 used for FIB-SEM milling (*top left*). TEM image of lamellae (200-nm thickness, 5 μm wide, scale bar: 5 μm) of Cr-PhotLOV1 crystals (*bottom left*) and diffraction image obtained from the lamella on a JEM F200 at 200 kV equipped with energy filter (0.7-μm beam size, 2197-mm detector distance). Diffraction images were collected with the help of Eric van Genderen (Laboratory of Multiscale Bioimaging, Paul Scherrer Institute, Switzerland).

**Figure 3:**
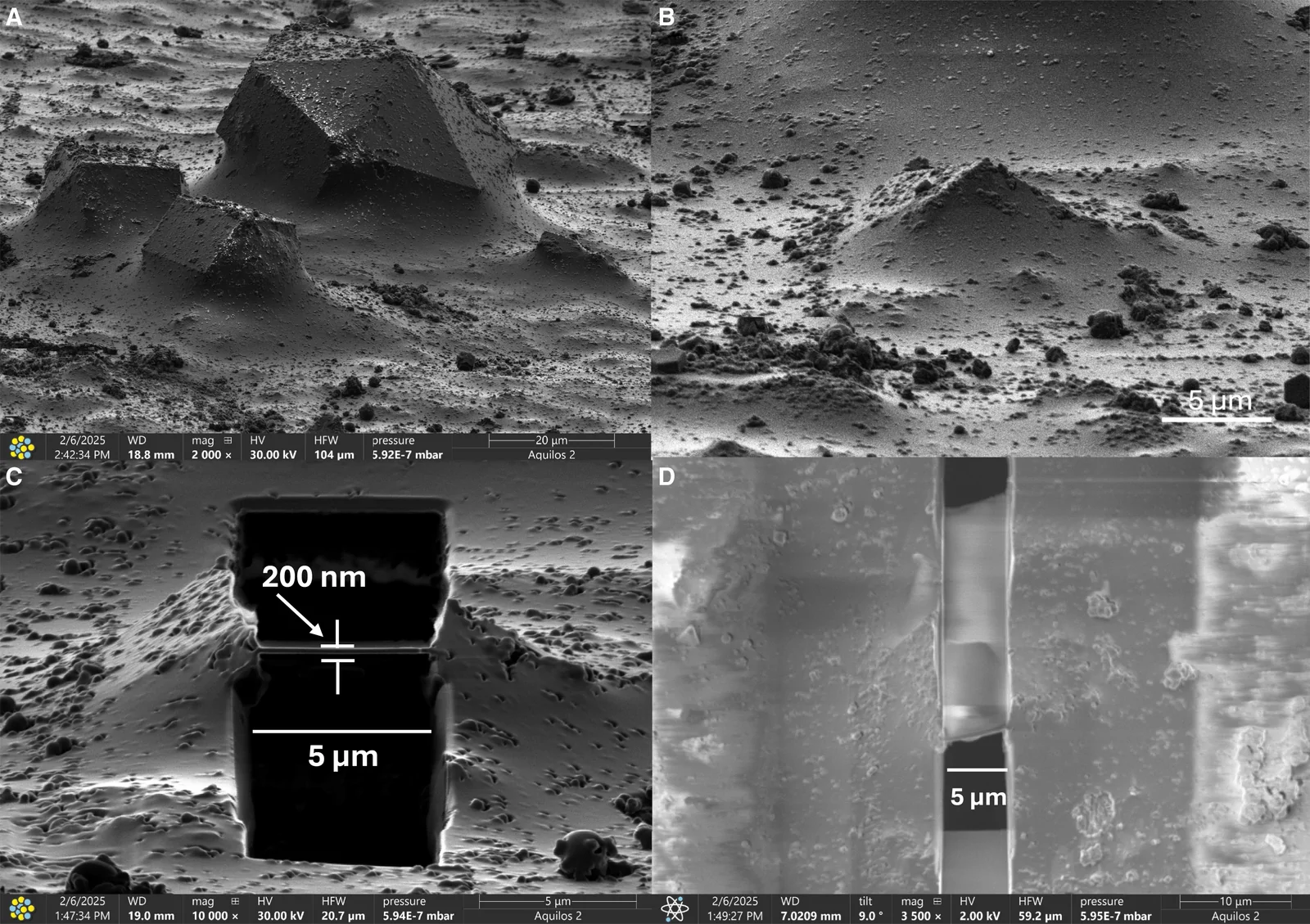
FIB-SEM milling of Cr-PhotLOV1 crysals. (*A*) Focused ion beam image of Cr-PhotLOV1 crystals on an EM grid. (*B* and *C*) Focused ion beam image of a Cr-PhotLOV1 crystal before, scale bar: 5 μm, and after milling with Ga ions, scale bars: 200 nm and 5 μm. (*D*) Image of the final Cr-PhotLOV1 lamella taken with the electron beam (200-nm thickness, 5 μm wide, scale bar: 5 μm). Milling was performed at the BioEM Lab of the University of Basel with the help of Moritz Hunkeler.

**Figure 4:**
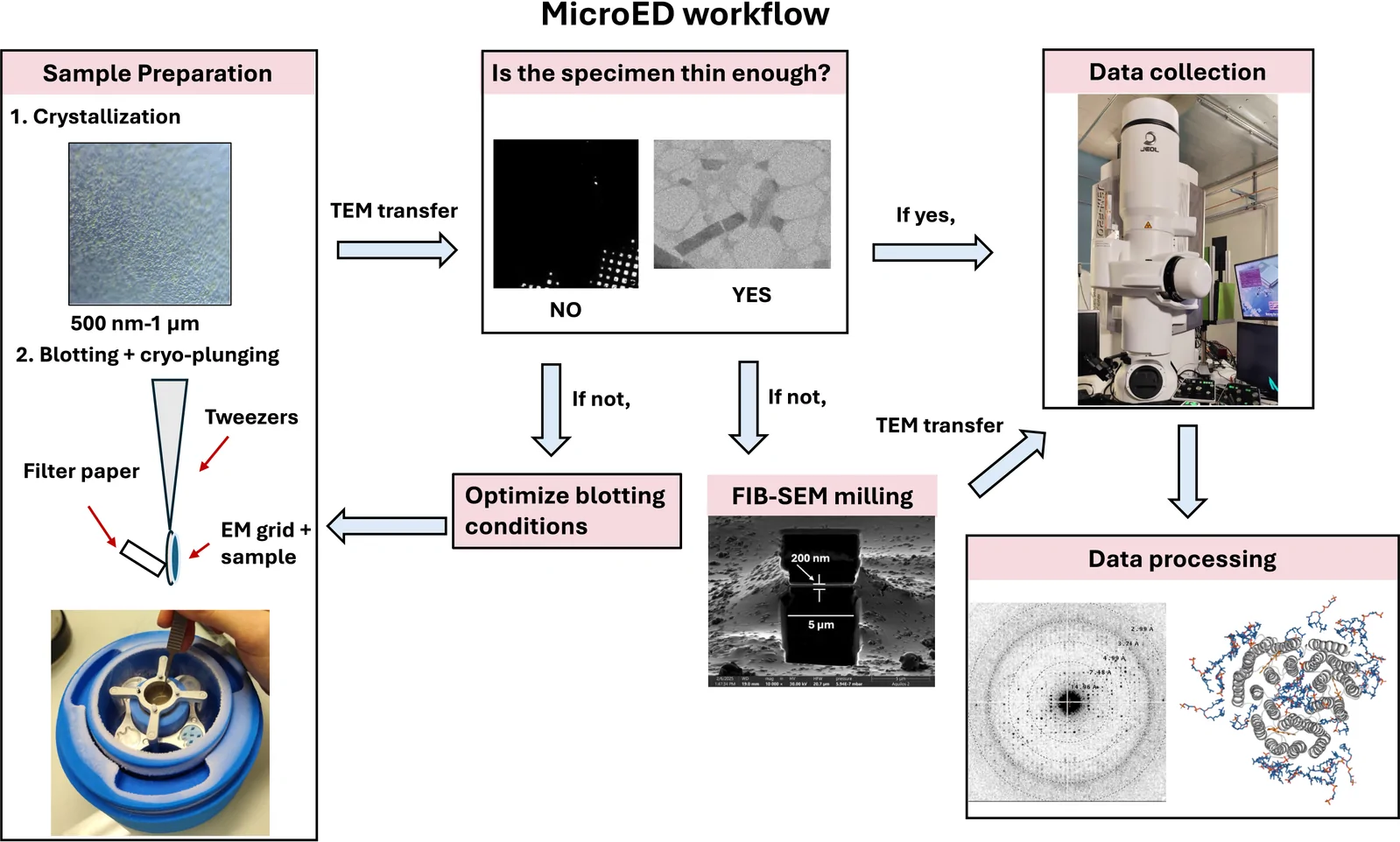
Overview of the MicroED workflow for protein samples. The sample is crystallized, blotted on an EM grid, and cryo-plunged in liquid ethane (note that for crystals grown in LCP, blotting as such is not possible due to the viscosity of the LCP). The quality of the sample can be assessed on the TEM. If the sample is not thin enough, it may be necessary to optimize further the blotting conditions (e.g., changing humidity, blotting force, or time). Alternatively FIB-SEM milling can be used to thin the specimen (e.g., for LCP samples). If the sample is thin enough, data collection can be performed on a TEM, working in diffraction mode. Data processing and structure refinement can be performed with software commonly available for x-ray data.

Importantly, when the protein does not spontaneously form nano-sized crystals, one can adjust the crystallization conditions to favor formation of smaller crystals (Fig. 2 *A*) (44,45). Alternatively, if crystals are not too fragile, they can be fragmented into smaller crystals by sonication, pipetting, or vortexing (43,46). Vigorous vortexing or pipetting are not doable for crystals of membrane proteins, which are generally a more challenging target to work with. If optimization of the crystallization conditions or fragmentation does not yield appropriately sized crystals, focused ion beam (FIB) milling can be used to produce thin lamellae of the crystals (Figs. 2 *B*, 3 *A*, and 4) (3,43,47).

An in-depth protocol for lamellae production using a gallium FIB has been previously reported (49). In summary, crystals are blotted on the carbon side of an EM grid before vitrification. Upon loading of the grid onto an FIB scanning electron microscope (FIB-SEM), equipped with a cryo-transfer, crystals are sputter coated with a layer of Pt (Fig. 3 *A*). It has been observed that FIB imaging causes damage on the surface of the crystals even at current values as low as 40 pA (47); however the Pt sputter coating step can significantly reduce the surface damage (47). Once the crystals of interest are located, milling is performed in three steps: rough milling (at relatively high beam currents of 100–500 pA), fine milling (beam currents of 30–100 pA), and polishing (beam currents of 1.5–30 pA). Notably, coating of the crystals before lamellae production and polishing of the lamellae have been proven to increase the lamellae survival and improve the quality of the data collection (50). Studies to benchmark the ideal thickness of the lamellae (47,51) suggest that the ideal thickness to obtain good diffraction data is between 100 and 300 nm for data collected on a TEM working at an accelerating voltage of 200 kV (Figs. 2 *B* and 3 *C*). Note that lamella deformation can be an issue as it increases mosaicity of the crystals (47), suggesting that care should be taken when performing MicroED data collection on lamellae.

The transfer of the crystals onto an EM grid can be challenging (e.g., when crystals are susceptible to damage). Moreover, some crystals may have a preferred orientation on the grid, thus limiting the achievable data completeness (3,52,53). Notably, when performing rotation of the crystal in a TEM, the rotation of the stage is physically limited to ±70° (54). An alternative to crystal transfer is the direct crystallization of the protein on the EM grid, namely suspended drop crystallization (52,53). However, FIB milling is still needed to remove the excess precipitant from the grid.

In a recent study, cryo-sectioning was used to prepare 150-nm-thick ribbons of lysozyme crystals that led to a 2.9-Å structure (55). Although cryo-sectioning could be an alternative method for when FIB-SEM access is not available, more studies are needed to assess the viability of the technique for other protein crystals.

After transfer of the crystals on an EM grid, the excess liquid needs to be blotted away to optimize signal/noise ratio while maintaining protein hydration (41) (Fig. 4). Automated systems such as Leica EM GP2 and Thermo Fisher Scientific Vitrobot enable to control blotting force, time, humidity, and temperature (43). Gravity cryo-plungers (56) offer control over temperature and humidity while blotting is performed manually. Blotting can be performed by touching either one or both sides of the EM grid with filter paper (43). The optimal method is sample dependent; however, one-sided blotting is generally preferred since it prevents crystals loss (43). Viscous samples, (e.g., with high percentage of PEG) are challenging to blot. To overcome such an issue, either dilution of the viscous buffer (43) or pressure-assisted blotting are possible options (41). Alternatives to blotting would be a direct sample transfer on the grid by picolitre lane writing and plasma dispersion on the grid using EasyGrid (EMBLEM) followed by an automatized vitrification process (57) or to use new automatic dispensing systems such as the cryoWriter (58). These blot-free methods may help reduce the risk of mechanically damaging the crystals.

After blotting, samples are vitrified in liquid ethane (43) (Fig. 4). Although sample-quality assessment can be performed directly on a TEM (or SEM), microscope time is valuable, and alternative screening methods are desirable. A recent study introduces an alternative method to assess vitreous ice layer thickness for EM grids before a TEM/SEM session (59). The setup uses an optical interferometric microscope equipped with a cryogenic stage and an image analysis software (59) that enable evaluation of the ice thickness on the EM grid.

## MicroED for membrane protein structure determination

The first example of membrane protein structure, determined by a combination of electron diffraction (ED) and imaging, comes from two-dimensional (2D) crystals of bacteriorhodopsin (bR) (12). This approach revealed the structure of bR within its endogenous lipidic environment. Subsequent improvements of the data collection and data analysis led to a near-atomic resolution structure of bR (3.5 Å, PDB: 1BRD and 2BRD) (60,61,62). This work guided the subsequent determination of LHC-II (13,14) and bacterial porin PhoE (63) structures, and it contributed to the Nobel Prize in Chemistry awarded to Richard Henderson in 2017 (64).

**Figure 5:**
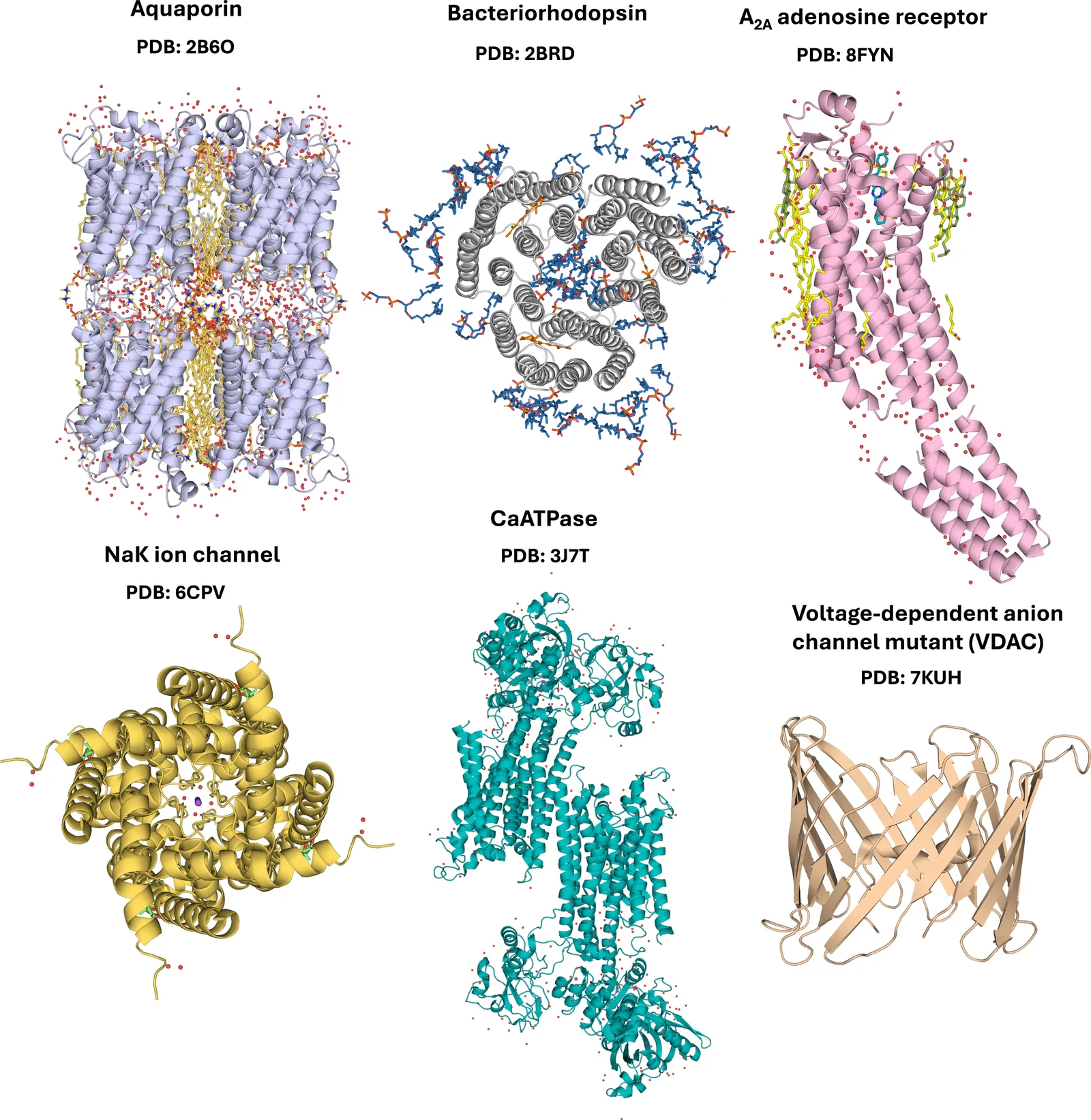
Overview of membrane proteins structures deposited on the PDB and solved by 2D electron crystallography or 3D ED/MicroED. Aquaporin, PDB: 2B6O; bacteriorhodopsin, PDB: 2BRD; A_2A_ receptor, PDB: 8FYN; NaK ion channel, PDB: 6CPV; CaATPase, PDB: 3J7T; mutant of the voltage-dependent anion channel, PDB: 7KUH.

In the early 2000s, the use of molecular replacement for structural determination enabled the determination of a junctional aquaporin (AQP0) structure, from 2D crystals, without the need to collect images of tilted specimens (65) (PDB: 2B6O, Fig. 5). Notably, the structure of the junctional AQP0 (PDB: 2B6O) differs from previously determined structures, as it is in a closed conformation and contains previously not observed pore constriction sites (65). AQP0 is reported to form junctions in vivo (65), and the junctional AQPO structure suggests that junction formation induces a closed conformation of the pore, with implications for the ion channel field and other aquaporins.

More recently, advances in ED sample preparation and data collection methods enabled successful determination of membrane protein structures from their 3D crystals, expanding the accessibility of the technique to samples that do not naturally form 2D crystals (6,32,66,67,68,69) (Fig. 5). MicroED is still in its infancy, so most reported studies serve primarily as proof of concept, demonstrating that the technique can be applied to membrane protein structure determination. Nevertheless, MicroED has also enabled the determination of previously unknown membrane protein structures, including vasopressin 1B receptor (66), a mutant of the voltage-dependent anion channel (70) and two new conformational states of the NaK ion channel (32,68,69) (Fig. 5). These are good indications that make us believe that, in the future, ED approaches will be highly appropriate for membrane protein crystals.

The structural investigation of membrane proteins presents several challenges, including low expression yield and laborious and expensive purification and crystallization processes (68). Although methods such as bicelle or lipidic-cubic phase (LCP) crystallization have been successful (68), the resulting crystals are often small and hard to optimize. Although XFELs can be used to study LCP-embedded crystals of membrane proteins (71), a method that minimizes sample consumption is preferable. When cryo-EM is not an option (e.g., membrane proteins with molecular weights <40 kDa), MicroED emerges as an alternative.

The high viscosity of the LCP makes blotting of membrane protein crystals challenging (71). Moreover, the presence of detergents or lipids surrounding the crystals can increase the background noise and negatively affect the signal/noise ratio (72).

A possibility is to directly transfer the crystals onto an EM grid and mill away the excess LCP layer, as previously done for bacteriorhodopsin (73). Direct transfer is, however, not always feasible (72). Moreover, small crystals that are buried in thick LCP are hard to localize on the grid (72). A possible solution is to fluorescently label the protein of interest and use an integrated fluorescence light microscope to locate the crystals on the grid (72), as previously described for human A_2A_ adenosine receptor (72). In a recent preprint (66), a similar approach was used to determine the previously unknown structure of vasopressin 1B receptor. In both studies a plasma FIB was used for milling the samples of interest.

Unlike gallium FIB, which make use of gallium ions, plasma FIB make use of plasma ion sources (e.g., xenon, oxygen, argon) (72,74). An initial characterization of the available plasma ion sources, performed on crystals of proteinase K (72) and lysozyme (74), showed that argon and xenon provide the highest milling rates while preserving lamellae integrity and provided the best data quality when compared with oxygen, nitrogen, or gallium (72,74). These results suggest that xenon and argon may be less damaging to protein crystals than the other tested ion sources (74).

There is added complication in LCP-generated crystals. The crystallites are always embedded in the LCP phase, and without removing this phase background scattering, ED would be unmanageable. Overall, FIB-SEM milling, particularly when combined with an integrated fluorescence light microscope arm, represents a powerful tool for preparing thin specimens of membrane protein crystals for MicroED experiments. As already demonstrated with the human A_2A_ adenosine receptor (72) and vasopressin 1B receptor (66), there is the possibility to overcome historical challenges in studying membrane protein structures and extending MicroED to other complex systems, such as retinal-binding proteins.

## MicroED data collection and processing

MicroED data collection of protein crystals can be performed on TEMs that operate in diffraction mode (25,75,76). Notably, for small-molecule MicroED, in addition to TEMs, dedicated electron diffractometers exist (Eldico Scientific or Rigaku) (25,26,27). These diffractometers have simplified hardware and software that offer some advantages over TEMs (e.g., a lack of imaging lenses removes potential issues arising from lens aberration or misalignment); however, the lack of a cooling system and fixed detector distances make these diffractometers not suitable for large unit cells such as those of protein crystals. Gonen et al. have previously provided a detailed description on how to set up a TEM to perform ED data collection of 3D crystals (54,75). In summary, after proper alignment of the beam, the EM grids loaded with the sample are screened at low dose rates (<10^−6^ e Å^−2^ s^−1^) and low magnification (100×) to identify areas with optimal ice thickness and crystal concentration (75). Once the region of interest is identified, crystals are inspected and centered and the eucentric height adjusted. Before data collection, it is advisable to insert selected area apertures and to configure the beam to the desired size to match the crystal size and minimize background noise. Diffraction data should then be collected with rapid scanning and at low doses (preferably at dose rates of 0.01–0.05 e Å^−2^ s^−1^) to minimize the radiation damage (75,77,78), and for this, sensitive fast detectors are key to achieve an optimal signal/noise ratio (79). Currently, different electron detectors can be used for MicroED data collection (79,80,81), including detectors commonly used for cryo-EM imaging (78,79); however electron counting detectors have been shown to produce relatively higher data quality (82). Data quality can be further improved with the use of energy filters (7) that reduce the background noise by removing inelastically scattered electrons based on their energy loss.

To increase the amount of the data obtainable out of a single nanocrystal, different data collection approaches have been investigated, and a more detailed description is provided by Gemmi et al. (38). In most of the published MicroED studies performed on proteins, rotation data collection was used (38). The rotation method is analogous to that used in x-ray diffraction (2,38,54). The EM grid containing crystals is continuously rotated while frames are being collected, and the angle of the rotation is generally limited by the stage to 60°–70° (83). Another possible approach is serial ED, where single diffraction patterns are taken by exposure of multiple crystals (84,85,86).

To accelerate MicroED data collection and make it viable to more users, automation is highly desirable. Several software packages for TEM data collection automation are currently available: SerialEM, ParalEM, EPUd, iTEM, eTasED, and Instamatic (87). However, the use of these software packages relies on their compatibility with the hardware of the TEM that is being used (87) and on the data collection approach required (83).

Data processing of MicroED data has been successfully performed using software developed for x-ray diffraction (54) (e.g., MOSFLM/AIMLESS, XDS, DIALS, and CrystFEL (84)), but accurate knowledge of the geometry of the measurements is required. Some of this information can be supplied when converting microscope images to a format compatible with the available processing pipelines (54,88). Alternatively, when processing with DIALS, a plug-in can be imported that reads special formats (88). A limited number of plug-ins are available for some of the cameras used for data collection (https://github.com/dials/dxtbx_ED_formats). However, due to the diversity of microscopes and detectors that are used for data collection, data conversion may currently require custom-made conversion tools and plug-ins. Notably, efforts are being made to establish standard data formats for MicroED and automated pipelines for data processing (89,90). Importantly, conventional x-ray crystallography software does not fully account for the specifics of ED data, such as the occurrence of multiple scattering events (91,92). By contrast, the recently developed PETS software was specifically designed to process ED data by incorporating dynamical diffraction effects and enabling accurate structure refinement from 3D data sets (91,92).

In MicroED, as in x-ray diffraction, the phase is lost (93). Due to the shorter wavelengths of electrons when compared with x-ray, anomalous scattering cannot be used to solve the phase (61,94). For high-resolution data, direct *ab initio* methods can be used to recover the phase (95). If a high-similarity model of the structure is available, or if the structure has been previously solved, data can be phased using molecular replacement in Phaser (96). For new structures, the molecular replacement model could be generated using AlphaFold (66,67,97,98,99); however, this requires some care, in particular when the accuracy of the predicted model is low. Other methods for phase recovery include imaging of the crystals (100) and radiation-induced damage (94). Finally, manual rebuilding and refinement can be performed with standard refinement packages developed for x-ray diffraction (54).

## Conclusion

Since the first concept of protein structure determination using ED in the 1970s (12) and the first structure of 3D crystals of lysozyme solved by ED in 2013 (101), ED has come a long way, and it has been used to solve numerous known and unknown protein structures (3,46,66,67,68,70,98,99,102). To date, more than 100 protein structures determined by ED have been deposited in the PDB. Of these, approximately 26 unique structures were obtained from 3D crystals or lamellae, and about six of them are membrane proteins. MicroED is also emerging as a potential tool for mechanistic studies, and it has been successfully used to trap a reaction intermediate forming during the reaction catalyzed by AgePgb protoglobin (97,103). Notably, efforts are undergoing to develop an ultrafast ED data collection method that would enable to study protein dynamics upon light activation of protein crystals (104).

With the advance of MicroED, more facilities are emerging to support sample preparation and data collection (25). Examples include eBIC, at Diamond Light Source UK, the UCSC cryo-EM facility, and The MicroED Core of UCLA in the USA. Moreover, ED has already been proven to be an important tool for the structural determination of small organic and inorganic molecules (9,33,34,35,36,37). Although challenges remain in applying this method to protein structure determination, particularly the need for automated data processing and, in some cases data collection, recent advances are laying the basis for broader accessibility. With continued innovation, MicroED is on its way to becoming a more widely adopted and more user-friendly technique.

## Acknowledgments

The authors acknowledge the 10.13039/100011963Swiss Nanoscience Institute, the Nano-Argovia program (grant 2024-03217 to V.P.), the ProtEDinNanoxtals consortium (ELDICO AG, leadXpro AG), Jan Peter Abrahams (Biozentrum, Basel, Switzerland), and Eric van Genderen (Paul Scherrer Institute, Villigen PSI, Switzerland) for supporting this project. The authors acknowledge Eric van Genderen for assistance with ED data collection of Cr-PhotLOV1 and Moritz Hunkeler and the BioEM Lab, Basel, for assistance with the FIB milling experiments. The authors acknowledge Guillaume Gotthard for discussion and input. The authors would like to thank Diamond Light Source for beamtime (bag proposal AP39) and the staff of eBIC, David Owen and Eilis Bragginton, for assistance with crystal testing, discussion, and input. Gebhard F.X. Schertler is supported by ERC-SOL grant 951644.

## Declaration of interests

Gebhard F.X. Schertler is a guest member of the Editorial Board of the *Biophysical Journal* for the special issue “Retinal proteins: Experiments and Computations.” He is a shareholder and advisor to leadXpro and InterAx.
